# Exploration of the Danggui Buxue Decoction Mechanism Regulating the Balance of ESR and AR in the TP53-AKT Signaling Pathway in the Prevention and Treatment of POF

**DOI:** 10.1155/2021/4862164

**Published:** 2021-12-30

**Authors:** Huaiquan Liu, Hong Yang, Zhong Qin, Yunzhi Chen, Haiyang Yu, Wen Li, Xing Zhu, Jingwen Cai, Jing Chen, Mengzhi Zhang

**Affiliations:** ^1^Shandong Xiandai University, Jinan, Shandong 250104, China; ^2^Guizhou University of Traditional Chinese Medicine, Guiyang, Guizhou 550025, China; ^3^Shanghai Shuguang Hospital Affiliated with Shanghai University of Traditional Chinese Medicine, Shanghai 201203, China; ^4^Shanghai Municipal Hospital of Traditional Chinese Medicine, Shanghai University of Traditional Chinese Medicine, Shanghai 200071, China; ^5^The Second Affiliated Hospital of Nanchang University, Nanchang, Jiangxi 330006, China

## Abstract

**Objective:**

The purpose of this study was to explore the molecular mechanism of Danggui Buxue Decoction (DBD) intervening premature ovarian failure (POF).

**Methods:**

The active compounds-targets network, active compounds-POF-targets network, and protein-protein interaction (PPI) network were constructed by a network pharmacology approach: Gene Ontology (GO) function and Kyoto Encyclopedia of Gene and Genome (KEGG) pathway analysis by DAVID 6.8 database. The molecular docking method was used to verify the interaction between core components of DBD and targets. Then, High-Performance Liquid Chromatography (HPLC) analysis was used to determine whether the DBD contained two key components including quercetin and kaempferol. Finally, the estrous cycle, organ index, ELISA, and western blot were used to verify that mechanism of DBD improved POF induced by cyclophosphamide (CTX) in rats.

**Results:**

Based on the network database including TCMSP, Swiss Target Prediction, DisGeNET, DrugBank, OMIM, and Malacard, we built the active compounds-targets network and active compounds-POF-targets network. We found that 2 core compounds (quercetin and kaempferol) and 5 critical targets (TP53, IL6, ESR1, AKT1, and AR) play an important role in the treatment of POF with DBD. The GO and KEGG enrichment analysis showed that the common targets involved a variety of signaling pathways, including the reactive oxygen species metabolic process, release of Cytochrome C from mitochondria and apoptotic signaling pathway, p53 signaling pathway, the PI3K-Akt signaling pathway, and the estrogen signaling pathway. The molecular docking showed that quercetin, kaempferol, and 5 critical targets had good results regarding the binding energy. Chromatography showed that DBD contained quercetin and kaempferol compounds, which was consistent with the database prediction results. Based on the above results, we found that the process of DBD interfering POF is closely related to the balance of ESR and AR in TP53-AKT signaling pathway and verified animal experiments. In animal experiments, we have shown that DBD and its active compounds can effectively improve estrus cycle of POF rats, inhibit serum levels of FSH and LH, protein expression levels of Cytochrome C, BAX, p53, and IL6, and promote ovary index, uterine index, serum levels of E_2_ and AMH, and protein expression levels of AKT1, ESR1, AR, and BCL2.

**Conclusions:**

DBD and its active components could treat POF by regulating the balance of ESR and AR in TP53-AKT signaling pathway.

## 1. Introduction

Premature ovarian failure (POF) is a disorder of the female reproductive system under the age of 40 years due to reduced ovarian function for various reasons. Generally, clinical manifestations include hot flashes, night sweats, hypaphrodisia, infertility, and persistent amenorrhea for more than 6 months [1]. There are several biochemical measurements that involve estradiol (E_2_), antimullerian hormone (AMH), follicular stimulating hormone (FSH), and luteinizing hormone (LH) to predict reproductive potential [2]. The global incidence of POF is approximately 1%, which is closely related to autoimmune diseases, neurological disorders, iatrogenic injuries, unhealthy living habits, etc. [3]. Currently, hormone replacement therapy (HRT) is mainly used for the treatment of POF. However, long-term treatment may easily cause anxiety, depression, irritability, and other adverse reactions. More seriously, it may even induce certain complications such as endometrial cancer, breast cancer, and unexplained vaginal bleeding [4]. What is noteworthy is that the traditional Chinese medicine (TCM) as complementary medicine therapy has not only achieved extremely effective results, but also greatly reduced the incidence of adverse reactions and complications [5].

Chinese herbal medicines and their derived secondary metabolism have different biological characteristics, which can prevent and treat a variety of human diseases [6], such as enhancing immunity, anti-inflammatory [7, 8], antiviral [9], regulation of oxidative stress [10], antianxiety [11], anticancer [12], and prevention of the genitourinary systems diseases [13]. It is worth noting that network pharmacology is an emerging branch of pharmacology composed of computer science, biology, medicine, and bioinformatics. It is usually committed to establishing network prediction models through public databases and high throughput to initially predict the molecular mechanism of drug treatment. Molecular docking is an important method for drug development and is used to study the interaction between receptor proteins and drugs. Therefore, the preliminary screening of targets and signaling pathways through the docking of network pharmacology and molecules and then using animal experiments for verification provide a promising way to quickly explore the pharmacological mechanism of traditional Chinese medicine.

### 1.1. Traditional Chinese Medicine

Gynecology believes that POF belongs to the categories of “blood depletion,” “amenorrhea,” and “infertility” and proposes that sufficient vital essence in the kidney is the basis for menstruation, and a deficient kidney essence would cause menstrual blood to dry up to form blood depletion. Miraculous Pivot indicates that women need sufficient blood to support themselves during menstruation and pregnancy; blood cannot fill the uterus and ovaries and other organs in the event of deficiency, resulting in amenorrhea and infertility. Danggui Buxue Decoction (DBD) is a classic recipe for nourishing “Qi” and enriching “Blood,” among which *Radix Astragali* can nourish yin and enrich blood, *Radix Angelicae sinensis* can promote blood circulation and nourish uterus. The combination of these two herbs can function together to enrich blood and nourish uterus. Modern medical studies have found that DBD and its main chemical constituents have a good therapeutic effect on women's reproductive problems such as POF, polycystic ovary syndrome (PCOS), irregular menstruation, and menopause [14, 15]. DBD has a series of pharmacological effects, including promoting hematopoiesis, increasing immunity, and regulating hormone levels [16]. DBD can increase the level of serum E_2_ and decrease the level of serum FSH and LH to improve hormone levels in ovariectomized (OVX) female rats and increase the weight of uterus [17, 18]. The serum of the drug in DBD can accelerate ovarian granular cell proliferation in rats and inhibit the activity of reactive oxygen species (ROS) free radicals to achieve the purpose of interfering with POF by reducing the protein expression level of the Caspase-3 [19].

Additionally, the pathogenesis of POF is still unclear and it is urgent to find its potential therapeutic targets. Therefore, this study mainly combines network pharmacology and molecular docking technology to explore potential targets and active compounds for DBD treatment of POF. The experiments in rats further explore the intervention mechanism of DBD on POF, which can provide important reference for the treatment of POF with TCM.

## 2. Materials and Methods

### 2.1. Network Pharmacology Analysis

#### 2.1.1. Screening of Active Compounds and Collection of Targets

The compounds were searched by inputting “Huangqi” and “Danggui” as keywords on Traditional Chinese Medicine Systems Pharmacology Database and Analysis Platform (TCMSP, https://tcmspw.com/tcmsp.php). Based on parameters of pharmacokinetics (ADME), oral bioavailability (OB) ≥ 30% and drug-likeness (DL) ≥ 0.18 were selected as the conditions for screening active compounds to determine the active compounds in DBD and obtain corresponding sequence of Canonical SMILES after further comparisons [20]. “Related Targets” in the TCMSP database and “Canonical SMILES” (the top 15 target targets were screened based on correlation) in the Swiss Target Prediction database (https://www.Swisstargetprediction.ch) were utilized to jointly predict the targets of active compounds [21]. All targets were introduced by the Uniprot database (https://www.Uniprot.org/), and then a network of “Active compound targets” was built with Cytoscape 3.7.2 software [22].

#### 2.1.2. Construction of Active Compound-POF-Targets Network

POF related targets were searched by entering “Premature ovarian failure” as keyword in DisGeNET (https://www.disgenet.org/), DrugBank (https://www.drugbank.ca), OMIM (https://omim.org/), and Malacard (https://www.malacards.org/) [23]. The Venn diagrams of the targets of DBD and POF were drawn out using R, and then the screening of common targets as important potential targets intervening POF in DBD was performed. The network of “active compound-POF-targets” was built using Cytoscape 3.7.2 software.

#### 2.1.3. Construction of Protein-Protein Interaction (PPI) Network

We introduced the targets intervening POF in DBD into STRING.11.0 (https://string-db.org/) platform where the mutual score was set as “medium confidence = 0.4” and the species was set as “Homo sapiens” in order to construct a PPI network [24]. The topological parameters of the PPI network were acquired by the “Network Analysis” function in the cytoscape3.7.2 software, among which the greater degree value is the most important in the PPI network. The top five core targets were selected based on degree scores as important targets for DBD to interfere with POF. Then, the PPI networks of the 5 core targets were constructed by the STRING.11.0 platform and setting “highest confidence = 0.9” and the species “Homo sapiens”.

#### 2.1.4. Biological Function and Pathway Analysis

The GO function and the analysis of the KEGG pathway were performed for POF targets that intervene in DBD and “Homo sapiens” species in the DAVID 6.8 database (https://david.ncifcrf.gov/home.jsp) [25]. The functions of the GO and KEGG pathways were subjected to visualization analysis using ggplot in the R package.

#### 2.1.5. Analysis of Molecular Docking between Active Compounds and the Core Protein Receptor

The top five targets of the degree value in the PPI network were selected as protein receptors, and the active compounds of the top 2 degrees were selected as ligands of the active compounds in the DBD intervening in POF for verifying the molecular docking, respectively. The target protein files in PDB format and the active compound files in SDF were obtained from the RSCB PDB database (https://www.rcsb.org/) and the PubChem database. Chem Office software was used to perform mol2 format conversion and energy minimization in active compounds. After water molecules, original ligands, and polarized hydrogen from target proteins were removed via PyMOL software, the affinity and binding energy between the target protein and the active compounds were analyzed by Autodock Vina and Python scripts. Meanwhile, the binding energy (affinity) ≤ −5.0 kJ/mol was a screening condition in this study [26].

### 2.2. Experimental Validation

#### 2.2.1. Drugs and Reagents

The *Radix Angelicae sinensis* granules (Cat:0119371) and *Radix Astragali* granules (Cat:0099211) are both produced by Guangdong Yifang Pharmaceutical Co., Ltd. According to clinical application, the approximate dose of DBD is *Radix Astragali* 30 g and *Radix Angelicae sinensis* 6 g, configured according to the ratio of *Radix Astragali* : *Radix Angelicae sinensis* = 5 : 1, and 2 g of *Radix Astragali* granules are equivalent to 10 g of *Radix Astragali* decoction pieces, and 3 g of *Radix Angelicae sinensis* granules are equivalent to 10 g of *Radix Angelicae sinensis* decoction pieces. We dissolved 6 g of *Radix Astragali* granules (equivalent to 30 g of *Radix Astragali* decoction pieces) and 1.8 g of *Radix Angelicae sinensis* granules (equivalent to 6 g of *Radix Angelicae sinensis* decoction pieces) each day in 50 ml of water solution to prepare a DBD solution with a crude drug concentration of 0.72 g/ml.

We used the following drugs and reagents: CTX (Shanxi Pude Pharmaceutical Co., Ltd., No. H14023686), quercetin, kaempferol (Chengdu Pusi Biological Technology Co., Ltd., Nos. PS012093 and PS011676), E_2_, AMH, FSH, LH (Wuhan Huamei Biological Engineering Co., Ltd., Nos. CSB-E05110r, CSB-E11162r, CSB-E06869r, and CSB-E12654r); GAPDH (Hangzhou Xianzhi Biological Co., Ltd., No. AB-P-R 001), HRP-labeled goat anti-rabbit secondary antibody (Wuhan Boster Biological Engineering Co., Ltd., No. BA1054), rabbit monoclonal antibody AKT1(Cell signaling, No. #75692), rabbit monoclonal antibody Cytochrome C (abcam, No. Ab133504), rabbit polyclonal antibodies ESR1, BCL2, BAX (Wuhan Sanying Biotechnology Co., Ltd., Nos. 21244-1-AP, 12789-1-AP, and 50599-2-lg), and rabbit polyclonal antibodies AR, p53, IL6 (Affinity, Nos. DF6783, AF0865, and DF6087).

#### 2.2.2. Chromatographic Analysis

The mixture of *Radix Astragali* granule 6 g and *Radix Angelicae sinensis* granule 1.8 g was dissolved in 50 ml ultra-pure water to make DBD. 1mg standard product of quercetin is dissolved in 1 ml methanol. 1mg standard product of kaempferol is dissolved in 1 ml methanol. Each sample was centrifuged at 4°C at 12000 rpm for 10 min. The instrument used in this experiment is the high-performance liquid chromatography, equipped with DAD detector. The chromatographic column was Diamonsil C18 (2) (250*∗*4.6 mm, 5 *μ*m), the flow rate was 1 ml/min, the column temperature was 35°C, the detection wavelength was 360 nm, the mobile phase was methanol : 0.1% phosphoric acid water/50 : 50, and the injection volume was 10 *μ*l.

#### 2.2.3. Animals

Fifty female Sprague Dawley (SD) rats of healthy specific pathogen-free (SPF) grade (8 weeks old, 190 ± 20 g) were purchased from Changsha Tianqin Biotechnology Co., Ltd. (animal license number: SCXK(Xiang)2019-0014). Rats were housed at a room temperature of 21 ± 2°C, humidity of 55 ± 5%, and normal light circadian rhythm, given a standard diet and distilled water, allowed to eat freely, and subjected to adaptive feeding for 7 days. The study was approved by the Institutional Ethics Committee of The Second Affiliated Hospital of Nanchang University and the examination and approval no. review [2021], no. (A709).

#### 2.2.4. Model and Administration

Fifty female SD rats were randomly divided into control group (*n* = 10), model group (*n* = 10), DBD group (*n* = 10), quercetin group (*n* = 10), and kaempferol group (*n* = 10). The POF model was induced in rats. First, 200 mg/kg CTX was injected intraperitoneally on the first day and then 8 mg/kg CTX for the following 14 days [27]. DBD (7.2 g/kg/d, crude drug concentration) was administered to rats in DBD group, quercetin (15 mg/kg/d) [28] to rats in quercetin group, and kaempferol (40 mg/kg/d) [29] to rats in kaempferol group for 4 weeks, while the rats in the control group and the rats in the model group received sterile distilled water by gavage (10 ml/kg/d) [30]. After 4 weeks of drug intervention, the estrus cycle was observed for 2 weeks, and then all rats were sacrificed.

#### 2.2.5. Evaluation of Estrous Cycles

Every day at 8:00 am, all rats were observed in the estrous cycle. Vaginal secretions were collected in 20 *μ*l 9% NaCl solution and smeared on the glass slide. After natural drying, soak in hematoxylin dye solution for dyeing for 5–10 min; rinse in running water; perform color separation with hydrochloric acid and ethanol for a few seconds; soak in tap water for 5 min; stain with eosin dye solution for 3 min; rinse in running water; dry, covered with slides; and microscopically inspect.

#### 2.2.6. Organ Index

After sacrifice, both the ovaries and uterine were removed surgically and weighed. The ovary index and uterine index were calculated: ovary index = the wet weight of bilateral ovaries (*g*)/body weight (*g*) × 100%, uterine index = the wet weight of bilateral uterine (*g*)/body weight (*g*) × 100%.

#### 2.2.7. ELISA

Optical densities (OD) were read at 450 nm and concentrations of E_2_, AMH, FSH, and LH were determined by comparison with standard curves.

#### 2.2.8. Western Blot

Ovary tissues were fully split by proteinlysis buffer. Then, supernatant was obtained after centrifugation. The sample protein was 50 *μ*g. Then, we performed gel preparation, electrophoresis, membrane transfer, and then blocking in 5% skim milk for 2 h. The membranes were incubated at 4°C overnight with anti-GAPDH (1 : 2000), AKT1 (1 : 1000), BAX (1 : 5000), Cytochrome C (1 : 5000), ESR1 (1 : 1000), IL6 (1 : 1000), p53 (1 : 1000), AR (1 : 1000), and BCL2 (1 : 2000). The next day, we wash the membranes, second antibody incubation at 37°C for 2 hours. The enhanced chemiluminescence (ECL) substrate developed in membranes. The result of the image was analyzed using ImageJ software.

#### 2.2.9. Data Analysis

All statistical analyses were executed with the SPSS 24.0 software program. Normal distribution was presented as mean ± standard deviation (SD). When the variance is homogeneous, LSD is used for pairwise comparison between groups. The comparison of results between multiple groups adopts one-way analysis of variance. When the data is abnormal distribution, the distributions were presented as median and interquartile ranges. The data were assessed with the nonparametric Kruskal–Wallis test, and the Nemenyi method was used to compare the two groups. All results were considered statistically significant with *P* < 0.05.

## 3. Results

### 3.1. Active Compounds and Targets of DBD

**Figure 1:**
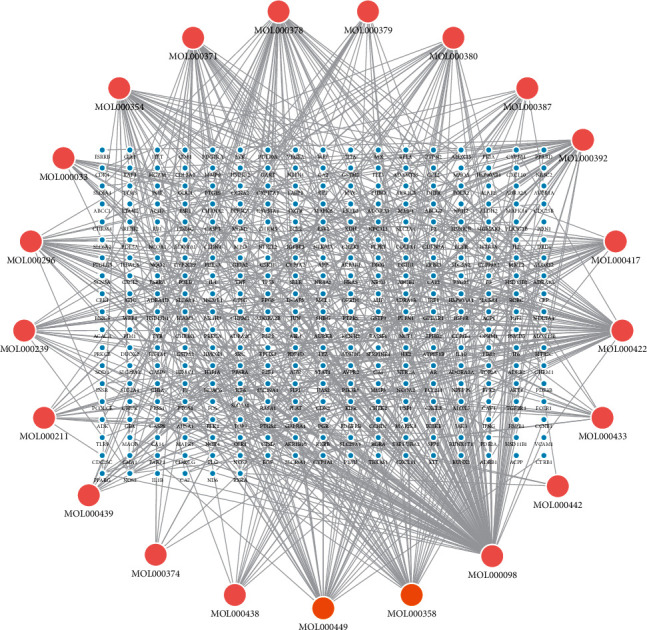
Active compounds-targets network diagram. Light blue represents targets, pink represents active compounds of *Radix Astragali*, and orange represents active compounds of *Radix Angelicae sinensis*.

22 active compounds in DBD were selected from the TCMSP database according to OB ≥ 30% and DL ≥ 0.18. After comparing with the PubChem database, MOL000398-isoflavanone was deleted because it does not have matching compounds and Canonical SMILES sequences of the remaining 21 active compounds were obtained. The target compounds in DBD were searched from TCMSP and Swiss Target Prediction databases where 183 and 155 corresponding targets were obtained, respectively, after identifying their names in combination with Uniprot database. 312 targets remained after deleting duplicates. Construction and analysis of the relationship between the active compound and the target in DBD were carried out by Cytoscape 3.7.2 software, and then we obtained the “active compound-target” network diagram, as shown in Figure 1. There are 333 nodes (312 targets, 21 active compounds) and 678 edges in the figure where circles in light blue represent the targets, pink represents the active compounds of *Radix Astragali*, and orange represents the active compounds of *Radix Angelicae sinensis*.

### 3.2. Potential Targets of DBD-Intermediating POF

**Figure 2:**
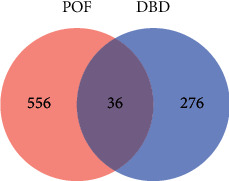
Venn diagram of DBD and the intersection genes in POF. DBD: Danggui Buxue Decoction; POF, premature ovarian failure.

160, 101, 99, and 366 known targets related to POF were searched from DisGeNET, DrugBank, OMIM, and Malacard databases, respectively. After deleting the duplicates, 592 POF-related pathogenic targets were obtained. Through the Venn diagram, the intersections of the active compound and the POF-related pathogenic 36 targets were obtained, as shown in Figure 2.

### 3.3. Active Compounds-POF-Targets Network

**Figure 3:**
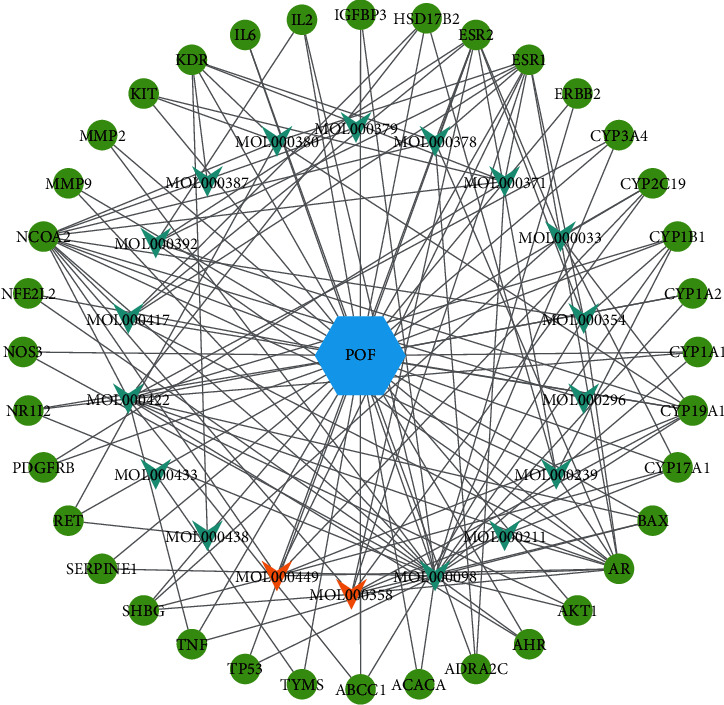
Active compounds-POF-targets network diagram. The light green circles represent targets, green V shapes represent *Radix Astragali*, orange V shapes represent *Radix Angelicae sinensis*, and the blue hexagon represents POF.

Related targets of intervening POF in DBD as well as the corresponding active compounds were inputted into Cytoscape 3.7.2 software to build “active compounds-POF-targets” network diagram, as shown in Figure 3. In the figure, there are 55 nodes (18 active compounds, 36 targets, and POF) and 143 edges, circles in light green represent the targets, V shapes represent the active compounds (green represents *Radix Astragali*, and orange represents *Radix Angelicae sinensis*), and the hexagon represents POF. After analysis of the mapping using the Network Analysis plug-in, it was found that the top 2 core active compounds with the degree value from high to low were MOL000098-quercetin and MOL000422-kaempferol. The topological parameters of the active core compounds mentioned above in the network of “active compounds-POF targets” are shown in Table 1.

### 3.4. PPI Network

**Figure 4:**
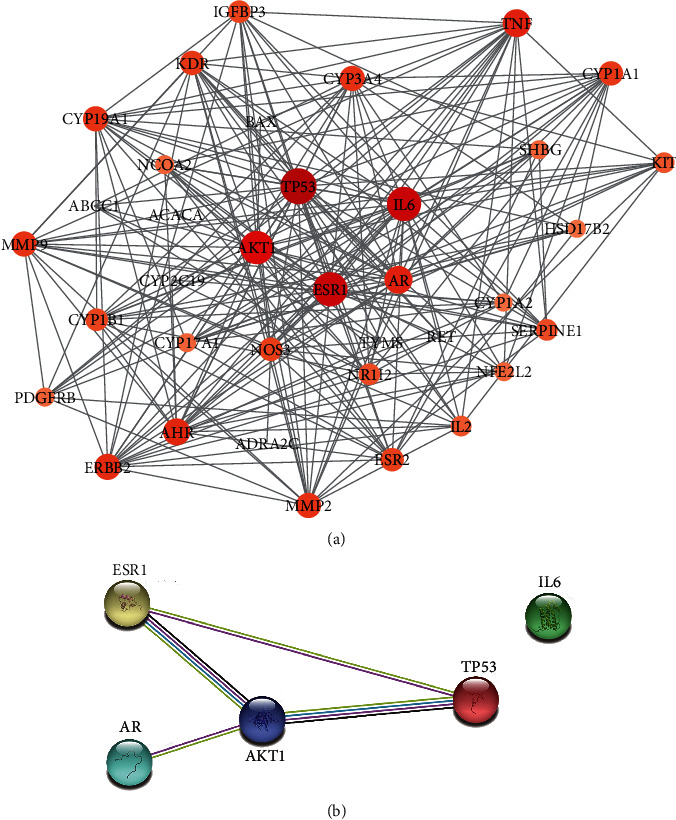
The PPI network of DBD-intermediating POF targets. (a) The PPI network of 36 targets of DBD intervening POF targets. The size and depth of the node color represent the scale of the degree value. (b) The PPI network of five core targets.

The protein -protein interaction (PPI) network was obtained from the STRING.11.0 database and redrawn by Cytoscape 3.7.2, as shown in Figure 4(a). There are 36 nodes (target protein) and 228 edges (protein interaction) in the PPI network diagram. The size and depth of the node color of the node represent the scale of degree value. The results of network topology analysis showed that five targets selected according to degree value were TP53, IL6, ESR1, AKT1, and AR, as shown in Table 2. Then we set “highest confidence = 0.9” in the STRING.11.0 database to build a PPI network of five core targets, as shown in Figure 4(b).

### 3.5. Biological Function and Pathway

**Figure 5:**
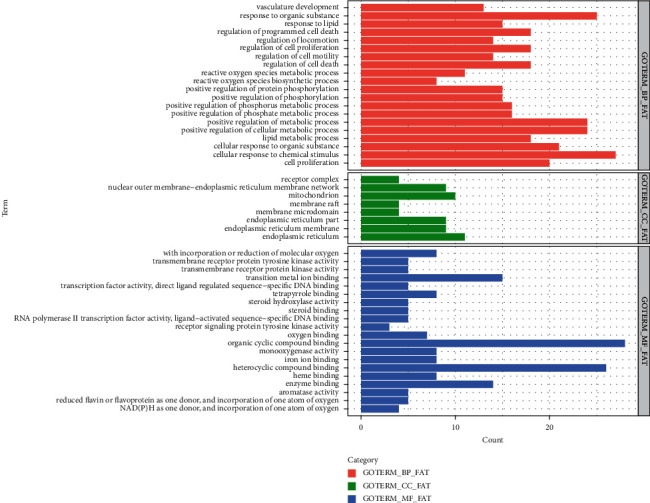
The histogram of the GO function of the POF targets mediated by DBD. The red indicates biological processes (BP), green indicates cell compositions (CC), and blue indicates molecular functions (MF).

The results of the analysis of the GO function showed that 919 enrichment results were obtained after screening (*P* < 0.05), including 829 biological processes (BP), 8 cell compositions (CC), and 82 molecular functions (MF). In the study, the top 20 BP, MF, and 8 CC results were screened as per *P*-value for mapping a histogram, as shown in Figure 5, where BP mainly covered reactive oxygen species metabolic process, release of Cytochrome C from mitochondria, and apoptotic signaling pathway, CC mainly included endoplasmic reticulum and mitochondrion, and MF generally were related to aromatase activity and steroid binding.

**Figure 6:**
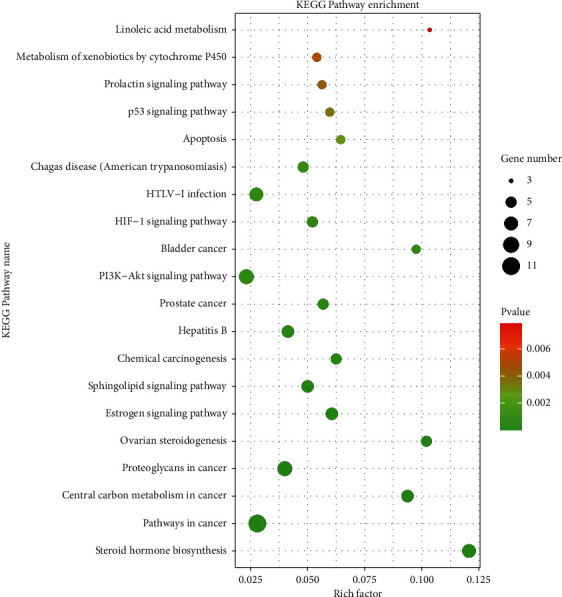
KEGG pathway bubble chart of DBD intervening POF targets.

KEGG analysis showed that 41 signaling pathways (*P* < 0.05) were involved in the p53 signaling pathway, PI3K-Akt signaling pathway, estrogen signaling pathway, ovarian steroidogenesis, etc. In the study, the top 20 signal pathways were selected according to the *P*-value to map a path bubble diagram, as shown in Figure 6.

### 3.6. Molecular Docking

**Figure 7:**
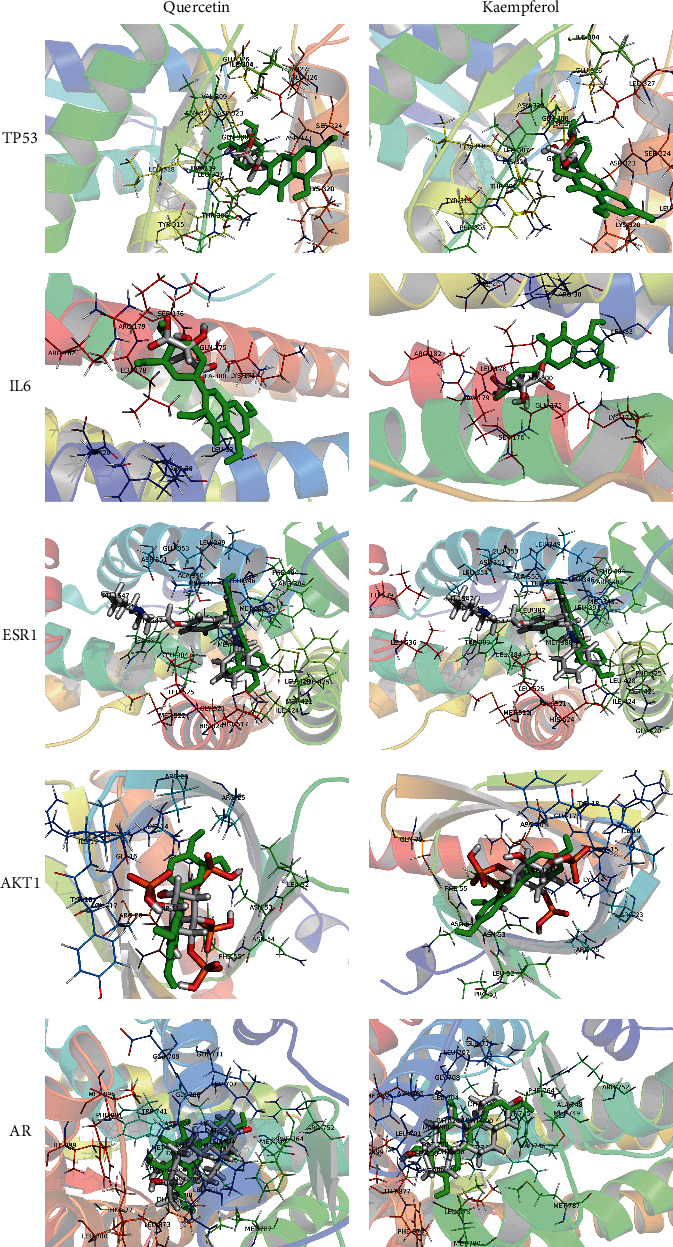
Molecular docking diagram of the core active compounds and the core targets.

The protein receptors and active compounds corresponding to TP53 (PID: 4CZ7), IL6 (PID: 1ALU), ESR1 (PID: 1UOM), AKT1 (PID: 1H10), and AR (PID: 1T73) were selected from the RSCB PDB database for molecular docking. The docking results of the 2 active core compounds and the 5 core protein receptors were all less than −5.0 kJ/mol, suggesting that the active core compounds in the DBD and core targets in POF had good binding capacity, as shown in Table 3 and Figure 7.

### 3.7. Chromatogram

**Figure 8:**
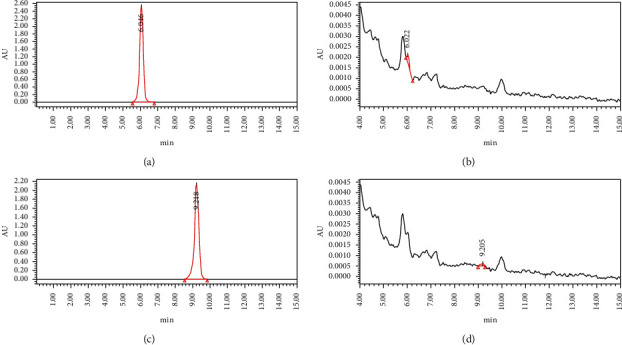
(a) Standard quercetin chromatogram. (b) Quercetin chromatogram in DBD. (c) Standard kaempferol chromatogram. (d) Kaempferol chromatogram in DBD.

In the chromatographic analysis, the retention time of the DBD sample is consistent with the retention time of the standard product, indicating that the sample contains two compounds, quercetin and kaempferol, as shown in Figure 8.

### 3.8. Estrous Cycles of Rats in Each Group

**Figure 9:**
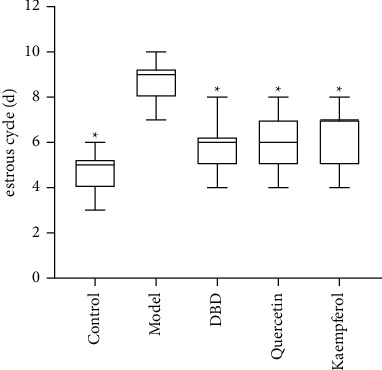
Effect of DBD on the estrous cycle in the rat POF model (*n* = 10). ^*∗*^*P* < 0.05, compared with model group.

Compared to control group, the estrus cycle of rats in the POF model group disordered. Compared to model group, estrus cycle in DBD group, quercetin group, and kaempferol group improved (*P* < 0.05), as shown in Figure 9.

### 3.9. Organ Index of Rats in Each Group

**Figure 10:**
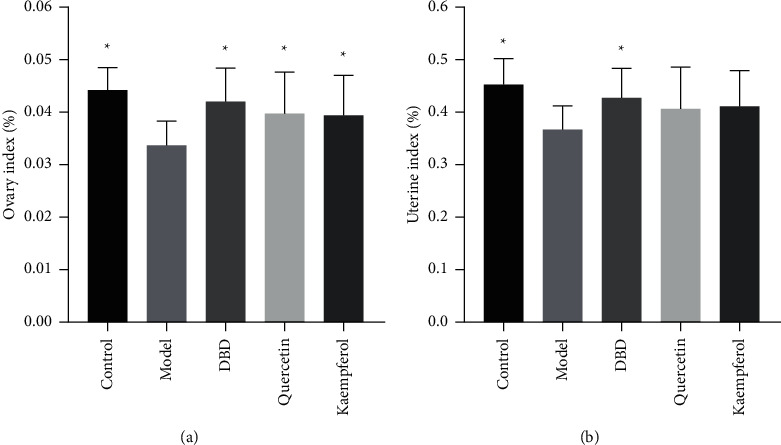
Effect of DBD on organ index in rat POF model. (a) Ovary index (*n* = 10). (b) Uterine index (*n* = 10). ^*∗*^*P* < 0.05, compared to the model group.

Compared to control group, the uterine index and the ovary index of rats in the POF model group decreased (*P* < 0.05). DBD group, quercetin group, and kaempferol group showed that ovary index increased compared to model group (*P* < 0.05) and DBD group showed that the uterine index increased compared to model group (*P* < 0.05), as shown in Figure 10.

### 3.10. The Level of E_2_, FSH, AMH, and LH in Serum

**Figure 11:**
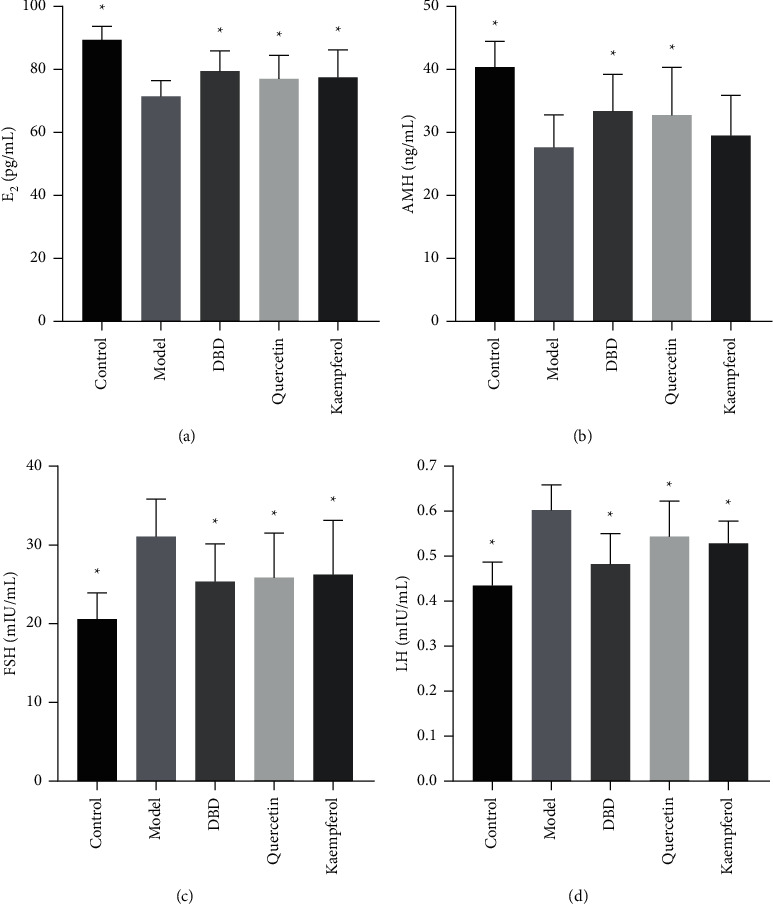
Effect of DBD on serum levels of E_2_, FSH, AMH, and LH in POF model rats. (a) Serum estradiol (E_2_) (*n* = 10). (b) Serum antimullerian hormone (AMH) (*n* = 10). (c) Serum follicular stimulating hormone (FSH) (*n* = 10). (d) Serum luteinizing hormone (LH) (*n* = 10). ^*∗*^*P* < 0.05, compared to model group.

Compared to control group, while the levels of E_2_ and AMH were significantly diminished, the levels of FSH and LH were significantly increased in the serum of model group rats (*P* < 0.05). Compared to model group, the levels of E_2_ were significantly increased, while the levels of LH and FSH were significantly diminished in rats serum of DBD group, quercetin group, and kaempferol group (*P* < 0.05) and the levels of AMH were significantly increased in the rats serum of DBD group and quercetin group (*P* < 0.05), as shown in Figure 11.

### 3.11. Protein Expression Levels of p53, IL6, Cytochrome C, BAX, AKT1, ESR1, AR, and BCL2 in Ovarian Tissue

**Figure 12:**
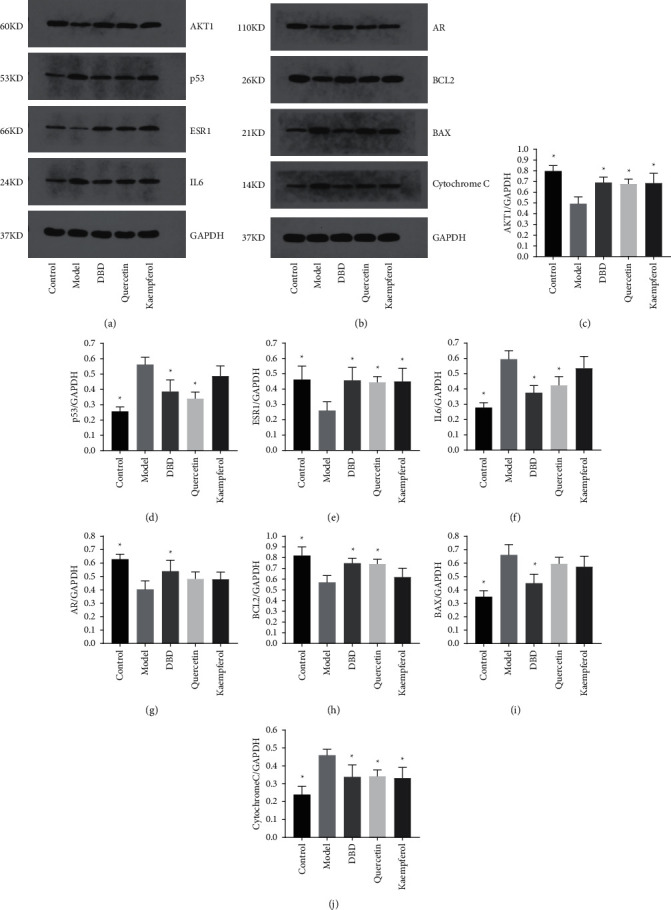
Effects of DBD on the expression of AKT1, p53, ESR1, IL6, AR, BCL2, BAX, and Cytochrome C proteins in ovary tissues. (a) Western blot analysis of the proteins AKT1, p53, ESR1, and IL6 proteins in the ovaries tissue. (b) Western blot analysis of the proteins AR, BCL2, BAX, and Cytochrome C in the ovary tissue; (c-j): Protein expressions of AKT1, p53, ESR1, IL6, AR, BCL2, BAX, and Cytochrome C (*n* = 3); ^∗^*P* < 0.05, compared to model group.

Compared to control group, the expression of p53, IL6, Cytochrome C, and BAX increased significantly, the expression of AKT1, ESR1, AR and BCL2 decreased significantly in the ovaries tissue of rats in the POF model group (*P* < 0.05). Compared to model group, the expression of AKT1 and ESR1 was significantly increased in DBD group, quercetin group, and kaempferol group, the expression of Cytochrome C was significantly decreased in DBD group, quercetin group, and kaempferol group, the expression of p53 and IL6 was significantly decreased in quercetin group and DBD group, the expression of BCL2 was significantly increased in quercetin group and DBD group, the expression of AR was increased significantly in DBD group, and the expression of BAX was decreased significantly in DBD group (*P* < 0.05), as shown in Figure 12.

## 4. Discussion

POF is a female reproductive system disease resulting from loss of ovarian function induced by autoimmune diseases, neurological disorders, iatrogenic injuries, bad lifestyle habits, and other factors. CTX exerts a certain toxicity on the gonad, accelerating the maturation of the primordial ovarian follicles into mature follicles. Meanwhile, long-term use or high-dose CTX can reduce the ovarian reserve in female patients [31]. Studies have shown that DBD could repair ovarian injury in rats by reducing the expression of ovarian CYP24A1 mRNA and increasing the expression of serum E_2_ and ovarian ERɑ mRNA [30]. DBD could have broad application prospects for the treatment of POF, and so it is of certain practical significance to carry out basic experimental research on it [32].

The analysis of the PPI network suggested that the process of intervening POF for DBD is highly correlated with TP53, IL6, ESR1, AKT1, AR, and other genes. As a gene related to senility, TP53 can induce the arrest of the development or apoptosis of the oocyte, thus accelerating the process of POF [33]. IL6 can not only induce B cell proliferation and T cell proliferation to produce an inflammatory response, but also increase the risk of POF complications and reduce the expression level of E_2_, testosterone (T), and ovarian volume (OV) [34]. The polymorphism at the ESR1 site affects the development process of POF, among which the polymorphism at the PvuII (rs2234693) site can induce many complications, such as osteoporosis and cardiovascular disease [35]. However, AKT1 phosphorylation is able to improve the fertility of POF model mice because it can promote the growth rate of secondary follicles and small follicles in the ovary and enhance the function of ovarian to secrete sex hormones [36]. By inducing the differentiation of primordial follicles into primary follicles, secondary follicles, and mature follicles by regulating AR, androgen is capable of regulating paracrine factors and reducing follicular atresia [37].

The GO and KEGG enrichment analysis showed that the reactive oxygen species metabolic process and release of Cytochrome C from mitochondria and apoptotic signaling pathway, p53 signaling pathway, the PI3K-Akt signaling pathway, and the estrogen signaling pathway play important roles in the intervention of DBD to POF. Persistent upregulation of ROS is the major feature of oxidative stress in cells and is an important factor causing cell aging [38]. Numerous studies have shown that GC apoptosis accelerates follicular atresia leading to POF [39]. A study has been shown to promote the release of Cytochrome C into the cytosol, which could lead to upregulation of cytochrome C in POF rat ovarian tissue [40]. The p53 signaling pathway can reduce the ovarian and uterine index and increase serum levels of FSH, LH, and the number of oocytes in the ovary by inhibiting the expression of Mdm2 and Mdm4 [41]. By regulating the PI3K/AKT signaling pathways, the sex hormone levels are effectively regulated, pathological injuries are relieved in POF rats, such as ovarian atrophy, cortical thickening, and structural disturbances, and the activation and maturation of ovarian granulosa cells are also promoted [42]. The estrogen signaling pathway is closely related to POF. Low estrogen level may cause estrous cycle disturbance and decreased ovarian function and spatial cognitive ability, as well as abnormal mental states in mice such as anxiety and depression [43]. The molecular docking results further verified the active compounds and potential targets predicted by network pharmacology and found that quercetin and kaempferol in DBD and the five core targets (TP53, IL6, ESR1, AKT1, and AR) have good results as regards the binding energy.

Based on the network database and bioinformatics results in this study, we found that the mechanism of DBD and its effective compounds in improving POF may be related to the balance of ESR and AR in the TP53-AKT signaling pathway. In the TP53-AKT signaling pathway, TP53 and AKT1 have the characteristics of mutual antagonists and play two opposite biological functions [44, 45]. TP53 can affect cell cycle arrest, apoptosis, and senescence by regulating the AKT pathway [46]. The balance of ESR and AR plays an important role in regulating the reproductive system, promoting ovarian development, and maintaining reproductive function [47]. Akt phosphorylation and inactivation can induce AR-dependent proliferative response and then participate in the process of regulating cell growth proteins [48]. The AKT can inhibit ESR-positive breast cancer cell proliferation [49]. Translocation of p-Akt may be related to suppression of estrogen-induced apoptosis in endometrial carcinogenesis [50]. DBD had a regulatory effect on the TP53-AKT signaling pathway and could inhibit the BAX/BCL2 ratio by activating Akt phosphorylation and estrogen receptors [51]. And by regulating BCL2 family proteins, DBD could inhibit cell apoptosis, promote the development of antral follicles, increase AMH secretion, and save ovarian function [32]. Quercetin and kaempferol, as the main active compounds of DBD, also have a certain therapeutic effect on common gynecological diseases. Quercetin is a pentahydroxy flavonol that could regulate the response to physical oxidative stress and the response to inflammation, increase the number of ovarian follicles and the level of estrogen expression in patients, improve the function of the ovarian reserve function, and gradually restore the patient's fertility [52]. Quercetin can also inhibit the proliferation of MCF-7 cells and attenuate mammary cancer by regulating TP53-AKT signaling pathway [53]. Kaempferol can regulate the levels of p53, AKT1, BAX, and BCL-X protein in the TP53-AKT signaling pathway of ovarian cancer cells, leading to apoptosis of ovarian cancer cells [54, 55].

Through the above situation, we used animal experiments to initially explore the regulation of DBD to regulate the balance of ESR and AR in the TP53-AKT signaling pathway, thereby preventing the molecular mechanism of treatment of POF. Research results show that DBD and its effective compounds can restore the estrus cycle, increase ovarian index, uterine index, serum E_2_, AMH levels, and ovarian AKT1, ESR1, AR, and BCL2 protein expression levels, reduce serum FSH and LH levels, ovarian p53, IL6, Cytochrome C, and BAX protein expression levels of POF model rats, and have a good therapeutic effect on POF. In comparison, DBD has the most comprehensive regulatory targets and the best effect, while quercetin and kaempferol have similar but different key targets in the treatment of POF. For example, the effect of quercetin and kaempferol on ESR1 is close to DBD. The effect of DBD on AR is better than quercetin and kaempferol. This suggests that traditional Chinese medicine and its effective compounds have their own advantages, and dialectical use may provide a better treatment for POF. This had also been verified in the research process of the treatment of asthma by *Schisandra chinensis* [56].

## 5. Conclusions

We found that DBD and its active components could treat POF by regulating the balance of ESR and AR in the TP53-AKT signaling pathway. However, due to the delay in updating the information recorded in the TCM, disease, and bioinformatics databases, certain critical targets or pathways can be overlooked. In the future, our teams will establish more studies in combination with transcriptomics, metabolomics, and proteomics to more fully explain the molecular mechanism of DBD during POF intervention.

## Figures and Tables

**Table 1:** The core active compounds with a high degree value in the active compounds-POF-targets network.

Compound	Degree	Stress	Topological coefficient
Quercetin	23	1980	0.1826087
Kaempferol	13	736	0.25128205

**Table 2:** Topological parameters of the core targets in PPI.

Gene symbol	Degree	Stress	Topological coefficient
TP53	28	676	0.39591837
ESR1	26	534	0.41758242
IL6	26	462	0.42527473
AKT1	25	604	0.40235294
AR	19	254	0.48297214

**Table 3:** The binding energy of core active compounds and core protein receptors.

Compound	Binding energy/(kJ/mol)				
	TP53	IL6	ESR1	AKT1	AR

Quercetin	−32.23	−27.21	−35.58	−24.70	−36.42
Kaempferol	−29.72	−25.53	−34.74	−24.28	−36.00

## Data Availability

The data that support the findings of this study are available from the corresponding author upon reasonable request.
